# Polymorphisms of XRCC4 are involved in reduced colorectal cancer risk in Chinese schizophrenia patients

**DOI:** 10.1186/1471-2407-10-523

**Published:** 2010-10-04

**Authors:** Yang Wang, Lei Wang, Xingwang Li, Baocheng Liu, Qingzhu Zhao, Peng Chen, Ti Wang, Tao Li, Jue Ji, Fengping Yang, Quan Wang, Jinfen Wang, Yanzeng Xiao, Yifeng Xu, Guoyin Feng, Zhihai Peng, Lin He, Guang He

**Affiliations:** 1 Bio-X Center, Key Laboratory for the Genetics of Developmental and Neuropsychiatric Disorders (Ministry of Education), Shanghai Jiao Tong University, 1954 Huashan Road, Shanghai 200030, China; 2 Institutes of Biomedical Sciences Fudan University, 138 Yixueyuan Road, Shanghai 200032, China; 3 Institute for Nutritional Sciences, Shanghai Institutes of Biological Sciences, Chinese Academy of Sciences, 320 Yueyang Road, Shanghai 200031, China; 4 Shanghai Institute of Mental Health, 600 South Wan Ping Road, Shanghai 200030, China; 5 Department of General Surgery, The Affiliated Shanghai First People's Hospital of Shanghai Jiao Tong University, 100 Hai Ning Road, Shanghai 200080, China; 6 Shanxi Cancer Hospital, 3 Zhi Gong Xin Street, Taiyuan 320013, China

## Abstract

**Background:**

Genetic factors related to the regulation of apoptosis in schizophrenia patients may be involved in a reduced vulnerability to cancer. XRCC4 is one of the potential candidate genes associated with schizophrenia which might induce colorectal cancer resistance.

**Methods:**

To examine the genetic association between colorectal cancer and schizophrenia, we analyzed five SNPs (rs6452526, rs2662238, rs963248, rs35268, rs2386275) covering ~205.7 kb in the region of XRCC4.

**Results:**

We observed that two of the five genetic polymorphisms showed statistically significant differences between 312 colorectal cancer subjects without schizophrenia and 270 schizophrenia subjects (rs6452536, p = 0.004, OR 0.61, 95% CI 0.44-0.86; rs35268, p = 0.028, OR 1.54, 95% CI 1.05-2.26). Moreover, the haplotype which combined all five markers was the most significant, giving a global *p *= 0.0005.

**Conclusions:**

Our data firstly indicate that XRCC4 may be a potential protective gene towards schizophrenia, conferring reduced susceptibility to colorectal cancer in the Han Chinese population.

## Background

The correlation between schizophrenia and cancer has been investigated for over a century, but the research on this epidemiological puzzle has produced contradictory findings [1,2]. According to recent studies, reduced risk and incidence for cancer were found to be associated with schizophrenia [3-5]. The explanations proposed for this finding have included genetic factors, neuroleptic medication, and environmental aspects [5-7]. The genetic basis of this lower risk has been ascribed to specific protective mechanism against cancer found in schizophrenia patients [7]. But until now p53 is the only gene to have been reported as being involved in this protective mechanism [8]. Recently, Park et al. [8] made a genetic association analysis of SNPs in the p53 gene between 104 lung cancer patients and 179 schizophrenia patients, and the results indicated that the p53 polymorphisms might be a genetic marker for lower susceptibility to lung cancer in schizophrenia patients.

The X-ray repair complementing defective repair in Chinese hamster cells 4 (XRCC4) gene is located on chromosome 5q14.2, which showed loss of heterozygosity (LOH) in sporadic colorectal cancer (Ratio 37.7%), and high ratio LOH indicates the presence of tumor suppressor locus. Moreover, in this region nearby allelic losses for tumor suppressor genes have been suggested as being associated with colorectal tumorigenesis [9]. The protein encoded by XRCC4 consists of 336 amino acid residues distributed among 8 exons, and has a long helical stem domain responsible for multimerization and interaction with DNA ligase IV [10]. By forming a complex with DNA ligase IV and DNA-dependent protein kinase, XRCC4 functions in the repair of DNA double-strand breaks (DSBs) by non-homologous end joining (NHEJ) and the completion of V(D)J recombination events [11].

The NHEJ pathway is required not only for normal development but also for suppression of tumors. Since it is one of the ubiquitous NHEJ components [12], XRCC4 might be considered as a potential tumor suppressor gene in several types of carcinoma. Disruption of XRCC4 in mouse embryonic cells leads to chromosomal instability, radiation hypersensitivity, and severely impaired V(D)J recombination [13]. In addition, Yan et al. have suggested that XRCC4 and, by extension, the NHEJ pathway is crucial for suppressing genomic instability in neuronal cells of mice [14]. Recently, Bau et al. have reported significant association of SNPs in the XRCC4 gene with colorectal cancer, indicating that the genetic polymorphisms of XRCC4 might be involved in colorectal carcinogenesis [15]. On the basis of the above evidence and the findings of Sugai et al., we deduced that XRCC4 might be involved in the development of colorectal carcinoma. Moreover, data from recent reports have revealed schizophrenia susceptibility loci on chromosome 5q14 [16,17], which is in the vicinity of the XRCC4 gene. In mice, XRCC4-deficiency leads to massive neuronal apoptosis [12].

We therefore concluded that variants within the XRCC4 gene might confer genetically reduced susceptibility to colorectal cancer among patients with schizophrenia. To examine this assumption, we investigated five genetic polymorphisms (rs6452526, rs2662238, rs963248, rs35268 and rs2386275) between Chinese colorectal cancer subjects without schizophrenia and schizophrenia subjects, a genetic association strategy similar to that used by Park et al. [8].

## Methods

### Subjects

A total of 312 sporadic colorectal cancer patients (178 male and 134 female, age 61.23 ± 14.03 years) and 270 schizophrenia patients (191 male and 79 female, age 57.25 ± 11.55 years) were recruited for this study. All the CRC patients underwent curative resection between 1999 and 2007 at the surgical department of the Shanghai First People's Hospital or the Shanxi People's Hospital, China. The cancerous tissue and adjacent normal control tissue (> 10 cm) were immediately frozen in liquid nitrogen. The pathologic tumor staging was performed according to Duke's criteria. The DSM-III-R was used as the diagnostic criterion for schizophrenia patients, all of whom were from Shanghai and were Han Chinese in origin. Two independent psychiatrists made a final diagnosis on the basis of interview data and hospital case notes. All subjects gave informed consent for the genetic analysis, which was reviewed and approved by the ethics committee of the Human Genetics Center in Shanghai. DNA was extracted using standard methods with phenol/chloroform purification.

### Genotyping

**Figure 1:**
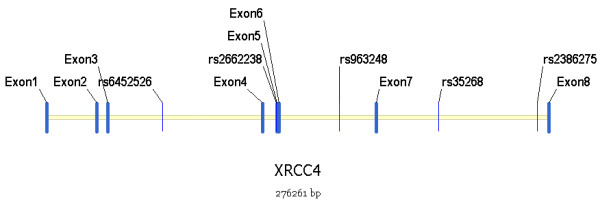
**The five SNPs selected among the genomic region of XRCC4**.

We genotyped five genetic polymorphisms, namely rs963248, which had been reported by Hayden et al. [11], and four other SNPs (rs6452526, rs2662238, rs35268 and rs2386275) from the HapMap project database http://www.hapmap.org and dbSNP http://www.ncbi.nlm.nih.gov/SNP/ to cover a ~205.7 kb region of XRCC4. All five markers are intronic SNPs (Figure 1). We genotyped these SNPs by the TaqMan^® ^assay method using the ABI 7900 DNA detection system (Applied Biosystems, Foster City, California). All probes and primers were designed by the Assay-on-Design service of Applied Biosystems. The standard PCR was performed using the Taqman^® ^Universal PCR Master Mix (Applied Biosystems) reagent.

### Statistical analysis

We analyzed Hardy-Weinberg equilibrium, allelic and genotypic distributions on http://202.120.31.137/myanalysis.php[18], a user-friendly platform which integrates efficient analysis tools for association studies. Monte Carlo simulation strategy and χ^2 ^test were used to compare the discrepancies of allele and genotype frequencies between CRC and schizophrenia patients [19]. We estimated linkage disequilibrium (LD) using "D" as the standardized measure, for all possible pairs of SNP loci. We corrected the *P *values of association analysis for multiplicity using a false discovery rate (FDR) controlling procedure [20]. Power calculations were performed using the G*Power program [21]. All the p values in the study were two-tailed and the significance level was set at *p = *0.05. Results were also expressed in terms of the odds ratio (OR) and 95% confidence interval (CI), which were calculated on the website http://www.hutchon.net/ConfidOR.htm. The program UNPHASED was used to estimate haplotype frequencies [22].

## Results

Genotype distributions of all five SNPs showed no significant deviations from Hardy-Weinberg equilibrium in either CRC or schizophrenia subjects. The allele and genotype frequencies of the SNPs are listed in Table 1. The power of rs6452526, rs2662238, rs963248, rs35268 and rs2386275 were of 0.767, 0.782, 0.852, 0.695 and 0.844 respectively, with an OR of 1.5 (95% CI). For genetic polymorphisms rs6452526, there were statistically significant differences in allele frequencies between the 312 CRC subjects and the 270 schizophrenia subjects (p = 0.004, p = 0.020, after the FDR correction). The T allele and TT genotype of rs6452526 were significantly less common in the CRC group compared to the schizophrenia group (allele, 13.4% versus 20.2%, OR 0.61, 95% CI 0.44-0.86; genotype, 2.3% versus 5.3%). Moreover, the allele frequency of rs35268 showed significant difference between the CRC and schizophrenia groups (p = 0.028, p = 0.070, after the FDR correction). The T allele of this marker was more common in CRC subjects than in schizophrenia subjects (90.9% versus 86.6%, OR 1.54, 95% CI 1.05-2.26).

**Table 1:** Allele and genotype frequencies of 5 SNPs among colorectal cancer and schizophrenia patients

SNP ID	genotype frequency(%)			*p *value*	FDR adjusted	allele frequency(%)		X^2^	*p *value*	FDR adjusted	Odds Ratio (95%CI)
rs6452526	CC	CT	TT			C	T				
CRC	194(75.5)	57(22.2)	6(2.3)	**0.021**	0.105	455(86.6)	69(13.4)	8.276	**0.004**	**0.020**	0.61(0.44-0.86)
SZ	159(64.9)	73(29.8)	13(5.3)			391(79.8)	99(20.2)				
rs2662238	AA	AG	GG			A	G				
CRC	12(4.3)	77(27.4)	192(68.3)	0.895	0.895	101(18.0)	461(82.0)	0.222	0.637	0.637	0.93(0.68-1.26)
SZ	13(5.0)	74(28.2)	175(66.8)			100(19.1)	424(80.9)				
rs963248	AA	AG	GG			A	G				
CRC	29(10.9)	105(39.6)	131(49.4)	0.296	0.493	163(30.8)	367(69.2)	1.303	0.254	0.317	1.17(0.89-1.53)
SZ	18(7.0)	105(41.0)	133(52.0)			141(27.5)	371(72.5)				
rs35268	CC	CT	TT			C	T				
CRC	2(0.7)	47(16.8)	230(82.4)	0.078	0.194	51(9.1)	507(90.9)	4.834	**0.028**	0.070	1.54(1.05-2.26)
SZ	3(1.2)	62(24.4)	189(74.4)			68(13.4)	440(86.6)				
rs2386275	AA	AG	GG			A	G				
CRC	124(52.3)	86(36.3)	27(11.4)	0.416	0.520	334(70.5)	140(29.5)	1.583	0.208	0.347	1.20(0.91-1.58)
SZ	146(56.2)	93(35.8)	21(8.1)			385(74.0)	135(26.0)				

*Pearson's *p *value, SNP = single nucleotide polymorphism, FDR = false discovery rate, CI = confidence interval, CRC = colorectal cancer, SZ = schizophrenia.

After analyzing linkage disequilibrium for each pair of the SNPs in the CRC and schizophrenia subjects (Table 2), two groups of markers (rs6452526-rs2662238 and rs963248-rs35268) were observed to be in strong LD respectively [23]. We therefore adopted the haplotype distributions for these SNPs in the later analysis. Haplotypes were omitted from analysis if the estimated haplotype probabilities were less than 3% in either the CRC or schizophrenia groups.

**Table 2:** Estimation of linkage disequilibrium between the 5 SNPs

	rs6452526	rs2662238	rs963248	rs35268	rs2386275
rs6452526		**0.99**	0.08	0.28	0.12
rs2662238	0.90		0.17	0.31	0.15
rs963248	0.00	0.00		**0.75**	0.42
rs35268	0.05	0.05	0.03		0.10
rs2386275	0.01	0.01	0.16	0.00	

For each pair of SNPs, *D*' > 0.7 are shown in boldface,

D' values are shown above and r^2 ^values below the diagoal,

SNP = single nucleotide polymorphism.

We selected only those haplotypes with significant frequency discrepancies between CRC and schizophrenia subjects (Table 3) for presentation. Haplotype analysis of these polymorphisms revealed some significant global p values (Table 4). One two-SNP-based haplotype and one five-SNP-based haplotype showed significant frequency difference between the CRC and schizophrenia groups. The haplotype combining all five markers was the most significant, giving a global *p *= 0.0005 (p = 0.0141, after the FDR correction). As its frequency was greater in the CRC group than in the schizophrenia group, the haplotype C-G-A-T-G (rs6452526-rs2662238-rs963248-rs35268-rs2386275) was found to be correlated with an increased odds ratio for CRC (*p *= 0.001, OR 2.07, 95% CI 1.32-3.24, p = 0.025, after the FDR correction). However, none of other significant haplotypes survived the FDR correction.

**Table 3:** Estimated haplotype frequencies and association significance

Haplotype*					Haplotype frequency(%)		X^2^	*p *value	Odds Ratio (95%CI)
			
rs6452526	rs2662238	rs963248	rs35268	rs2386275	CRC	SZ			
T	A				63.97(13.0)	98.00(20.2)	8.251	0.004	0.61(0.43-0.86)
C	G				415.97(84.2)	386.00(79.8)	8.251	0.004	1.65(1.17-2.33)
		G	C		40.64(8.1)	61.29(12.5)	5.118	0.024	0.62(0.41-0.94)
T	A	G	T	A	18.46(5.2)	45.45(10.2)	6.693	0.010	0.48(0.28-0.85)
T	A	G	C	A	5.22(1.5)	20.07(4.5)	5.918	0.015	0.32(0.12-0.84)
C	G	A	T	G	54.49(15.2)	36.21(8.2)	10.445	0.001	2.07(1.32-3.24)

*Haplotypes were omitted from analysis if the estimated haplotype probabilities were less than 5%

CI = confidence interval, CRC = colorectal cancer, SZ = schizophrenia.

**Table 4:** Global p values of estimated haplotypes of the 5 SNPs within XRCC4

Haplotype	Global *p *value*	FDR adjusted
rs6452526-rs2662238	**0.0041**	**0.0212**
rs963248-rs35268	0.0599	0.0708
rs6452526-rs2662238-rs963248-rs35268-rs2386275	**0.0005**	**0.0141**

*Pearson's *p *value, FDR = false discovery rate, statistical significance set at *p *< 0.05,

SNP = single nucleotide polymorphism.

In the power calculations using the G*Power 3 program, our sample size had greater than 90% power to detect a significant (α < 0.05) association for alleles, genotypes and haplotypes when an effect size index of 0.1 (corresponding to a "weak" gene effect) was used.

## Discussion

The question whether schizophrenia is linked to a decreased risk of developing cancer has been the subject of considerable research over a number of decades. The debate has centered on the incidence of malignancies in patients with schizophrenia which have been variously reported to be higher, lower, or similar to that in the general population. However, the majority of studies in the last decade have suggested that schizophrenia patients are protected against cancer in general [3-5].

Several possible explanations have been offered for the evidence that cancer risk and incidence are reduced in patients with schizophrenia. In one of the earlier studies, Mortensen observed a lower risk of prostate cancer among schizophrenia patients who had been exposed to prolonged neuroleptic medication [6]. More recent theories have focused on the possibility that increased apoptosis could account for the neurodevelopmental abnormalities as well as tumor resistance linked to schizophrenia [7], an explanation supported by Park et al. in a Korean cohort [8]. XRCC4 encodes a nuclear phosphoprotein that multimerizes and interacts with DNA Ligase 4 and DNA-dependent protein kinase, playing a critical role in the non-homologous end joining (NHEJ) pathway [11]. In mice, XRCC4-deficiency is embryonically lethal with a massive neuronal apoptosis, and XRCC4 has been observed to interplay with p53 in the regulation of apoptosis, indicating that XRCC4 is crucial for maintaining genomic stability and for the suppression of tumors [12]. Thus, XRCC4 might be a potential candidate gene for the hypothesis that schizophrenia offers reduced susceptibility to malignancy.

In the present study, our data based on Han Chinese samples provide further support for the assumption that XRCC4 might be involved in a potential protective mechanism against cancer in schizophrenia patients. We conducted the genetic analysis by genotyping five SNPs, including one SNPs previously investigated by Hayden et al. [11], and four other SNPs selected from the HapMap project database http://www.hapmap.org and dbSNP http://www.ncbi.nlm.nih.gov/SNP/. At two of the five markers (rs6452526 and rs35268) there were statistically significant discrepancies of allele or genotype frequencies between CRC and schizophrenia subjects. We observed that the T allele of r6452526 was less frequent in CRC than in schizophrenia subjects (OR 0.61, 95% CI 0.44-0.86, p = 0.004, p = 0.020, after the FDR correction), indicating that it might be a protective factor for schizophrenia patients against CRC; whereas the higher frequency of the T allele for rs35268 in CRC than in schizophrenia subjects (OR 1.54, CI 1.05-2.26, p = 0.028, p = 0.070, after the FDR correction) implies that the T allele might be a risk allele for CRC.

Since haplotypes constructed from closely located markers will typically increase the statistical power for association with the disease, we performed haplotype analysis in SNPs with strong linkage disequilibrium (D' > 0.7). Our results indicated that one two-SNP-based haplotype and one five-SNP-based haplotype showed significant global frequency difference between the CRC and schizophrenia groups (Table 4). The most significant window spanned all five SNPs giving a global p = 0.0005 (p = 0.0141, after the FDR correction). In addition, we observed that the most significant haplotype C-G-A-T-G (rs6452526-rs2662238-rs963248-rs35268-rs2386275, OR 2.07, 95% CI 1.32-3.24, p = 0.001, p = 0.025, after the FDR correction) was twice as common in the CRC group (15.2%) as in the schizophrenia group (8.2%), suggesting that C-G-A-T-G is a risk haplotype for CRC. In addition, we have compared the frequencies of C-G-A-T-G haplotype between CRC patients and normal controls, but the individual P value showed there was no difference.

Park et al. using genetic association analysis, showed that p53, as a protective gene, induced a lower incidence of lung cancer among Korean patients with schizophrenia [8]. Using a similar research strategy, we found that polymorphisms of XRCC4 may confer genetically reduced susceptibility to CRC among Chinese schizophrenia patients. Compared to the study of Park et al. our sample was twice as large and involved better age-matched subjects, thus decreasing the possibility of false-positive results [24]. However, the genetic contribution to this reduced risk for CRC among schizophrenia subjects is likely to involve a series of susceptibility loci, each influencing but not determining overall risk. In addition, our sample size is relatively small, thus additional replication studies using more SNPs in large non-Asian samples are needed. Genetic, environmental, and pharmacological influences are all thought to be contributing factors, which poses a major challenge for solving this epidemiological puzzle [5-7].

## Conclusions

In summary, our results provide a first indication that XRCC4 might be a potential protective gene with respect to schizophrenia, conferring decreased susceptibility to colorectal cancer in the Han Chinese population. Genetic studies to date have focused on subjects of Asian ethnicity, and further research needs to be undertaken in other ethnic groups. Replicating studies with more markers and with larger samples will be necessary to clarify the correlation between schizophrenia and cancer.

## Competing interests

The authors declare that they have no competing interests.

## Authors' contributions

Author Yang Wang designed the study, managed the literature searches, and wrote the first draft of the manuscript. Authors Zhihai Peng, Lin He and Guang He undertook the statistical analysis. Authors Lei Wang, Peng Chen, Ti Wang, Tao Li, Jue Ji, Fengping Yang, Guoyin Feng and Xingwang Li performed genotyping and collected the data. Authors Baocheng Liu, Qingzhu Zhao, Quan Wang, Jinfen Wang, Yanzeng Xiao and Yifeng Xu conducted the clinical studies and literature search. All authors contributed to and have approved the final manuscript.

## Pre-publication history

The pre-publication history for this paper can be accessed here:

http://www.biomedcentral.com/1471-2407/10/523/prepub
